# The Type III Accessory Protein HrpE of *Xanthomonas oryzae* pv. *oryzae* Surpasses the Secretion Role, and Enhances Plant Resistance and Photosynthesis

**DOI:** 10.3390/microorganisms7110572

**Published:** 2019-11-18

**Authors:** Taha Majid Mahmood Sheikh, Liyuan Zhang, Muhammad Zubair, Alvina Hanif, Ping Li, Ayaz Farzand, Haider Ali, Muhammad Saqib Bilal, Yiqun Hu, Xiaochen Chen, Congfeng Song, Hansong Dong, Meixiang Zhang

**Affiliations:** 1 Department of Plant Pathology, College of Plant Protection, Nanjing Agricultural University, Nanjing 210095, China; tahamajid1705@yahoo.com (T.M.M.S.); zubair_biotech@yahoo.com (M.Z.); rao.alvina@yahoo.com (A.H.); 2016202014@njau.edu.cn (P.L.); ayaz.farzand@uaf.edu.pk (A.F.); haiderali.karyala@gmail.com (H.A.); 2018202066@njau.edu.cn (M.S.B.); 2015202002@njau.edu.cn (Y.H.); 2017202001@njau.edu.cn (X.C.); cfsong@njau.edu.cn (C.S.); 2 Department of Plant Pathology, Shandong Agricultural University, Taian 271018, China; lyZhang@sdau.edu.cn; 3 State Key Laboratory of Crop Biology, Taian 271018, China; 4 Department of Plant Pathology, University of Agriculture, Faisalabad, P.O. Box 38040, Pakistan

**Keywords:** HrpE, type III secretion system, effector secretion, virulence, immunity

## Abstract

Many species of plant-pathogenic gram-negative bacteria deploy the type III (T3) secretion system to secrete virulence components, which are mostly characteristic of protein effectors targeting the cytosol of the plant cell following secretion. *Xanthomonas oryzae* pv. *oryzae* (*Xoo*), a rice pathogen causing bacterial blight disease, uses the T3 accessory protein HrpE to assemble the pilus pathway, which in turn secretes transcription activator-like (TAL) effectors. The *hrpE* gene can execute extensive physiological and pathological functions beyond effector secretion. As evidenced in this study, when the *hrpE* gene was deleted from the *Xoo* genome, the bacteria incur seriouimpairments in multiplication, motility, and virulence. The virulence nullification is attributed to reduced secretion and translocation of PthXo1, which is a TAL effector that determines the bacterial virulence in the susceptible rice varieties. When the HrpE protein produced by prokaryotic expression is applied to plants, the recombinant protein is highly effective at inducing the defense response. Moreover, leaf photosynthesis efficiency is enhanced in HrpE-treated plants. These results provide experimental avenues to modulate the plant defense and growth tradeoff by manipulating a bacterial T3 accessory protein.

## 1. Introduction

Bacteria adopt different life styles for adapting to their surroundings; alone, cooperative or synergetic, commensal and parasitic. Bacterial cells utilize their flagella to swim and look for a better environment for their survival [1]. The interactions among gregarious and extroverted cells with their host tissues are established by means of pili or fimbriae, and colonies and biofilm formation for their survival [2,3]. Pathogenic bacteria adhere to the eukaryotic cells, which is considered an important step in effective colonization of the host tissue. There are certain structures present on the bacterial cell surface that are involved in the adhesion of bacteria, including a vast group of fimbrial and non-fimbrial adhesions. Fimbrial adhesions include pili, which are hair-like appendages present on the surface of most of the bacteria. However, they are different in the mechanism of their assembly, structure and function [4]. Bacteria are thought to use various types of secretion systems to translocate the toxins and effector proteins from the source to the sink [5]. There are six different types of secretion systems (type I to type VI) that have been reported to be responsible for substrate secretion in gram-negative bacteria. Out of these, the type III (T3) secretion system plays a predominant role in secretion of toxins and proteic effectors that makes them virulent against their host [6].

In *Xanthomonas*, a chromosomal region of 23 kb length contains the hrp gene cluster where it is organized into 6 different operons. The harpins included in the hrp gene cluster are nominated as *hrpA* to *hrpF* [7]. These two proteins are substrates of the T3 secretion system where they play a predominant role in the delivery of T3 proteic and toxic effectors through and towards the plasma membrane of the plant cell. The *hrpE* gene encrypts the HrpE protein of 9 kDa size, which formulates a slender pilus with a length and diameter of 4 µm and 8 to 10 nm, respectively. However, the Hrp pilus acts as an appendage of the cell surface in the T3 secretion system. On the basis of various logics including mutational analyses and electron microscopy, HrpE was considered to be the structural constituent of the hrp pilus, which is made up of pilin subunits while HrpA is regarded as the best hrp pilus subunit [8]. Mutations in the hrp genes of bacteria affect the virulence, so they neither produce disease in susceptible hosts nor elicit a hypersensitive response (HR) in resistant plants [8,9]. A *hrpA* mutant from *Pseudomonas syringae* DC3000 failed to elicit a HR despite the fact that the bacteria had *avr* gene, which interact with an R gene in the plant [9]. In *Xanthomonas citri pv. citri*, biofilm formation and motility of the bacteria was affected by the deletion of the *hrpB* gene [10]. Gene deletion from the genome of *Riemerella anatipestifer* affected the bacterial virulence, gene expression and growth patterns in liquid media [11].

The T3 secretion system is extremely conserved in pathogenic gram-negative bacteria including plant pathogens. *Xanthomonas oryzae pv oryzae* (*Xoo*) is a gram-negative bacterial plant pathogen that causes a disease named bacterial blight in rice [12]. The plant and bacteria undergo several layers of interactions to confer plant susceptibility (disease) or insusceptibility (resistance), as a common feature of pathogenesis. The first interaction that occurs between the pathogens and their hosts takes place through a structure called a pilus [13]. The pilus is responsible for bacterial effectors and protein translocation from the two bacterial membranes into the interior of the host cell, by the trafficking across the plasma membrane of the host and in some circumstances across the cell wall of the plants [14]. The structure of the pilus is almost similar to that of the flagella, but additionally has the cap formed on the tip during assembly [5]. Almost ten T3 secretory proteins are similar in membrane topology and sequence to the flagellar proteins [15]. Although the pilus and flagella share some functions, the pilus is distinct from the flagella in regards to induction of T3 secretion by host sensing and the ability to translocate the proteins into eukaryotic cells [3]. The HrpE protein in *Xanthomonas* species and pathovars is highly conserved, including *X. campestris pv. vesicatoria, X. citri pv. citri* and *X. campestris pv. campestris* [16]. Therefore, HrpE assembly into the pilus is crucial for effector secretion by these bacteria. Transcription activator-like (TAL) effectors play essential roles in the interactions between *Xanthomonas* and their hosts. Several TAL effectors from *Xoo* have been reported to be major virulence factors that may activate the expression of susceptibility genes (*S*) during infection. The key virulence factor of the *Xoo* strain PXO99^A^ in rice is PthXo1 [17].

Harpins have been characterized to elicit disease resistance through the NIM1 (non-inducible immunity)-mediated SAR (systemic acquired resistance) signal transduction pathway. The action site of harpin is upstream of salicylic acid (SA) in the SAR regulatory pathway [18]. Moreover, harpins have been considered as a potential component of pathogen associated molecular patterns (PAMPs) based on studies demonstrating the role of T3 accessory proteins in inducing PAMP-triggered immunity (PTI) in plants externally treated with these proteins [18,19,20,21]. PTI is activated once a PAMP molecule is recognized by one of the pattern recognition receptors situated in the plasma membrane of plant cells [22]. A physical role of PTI is to limit the growth of the pathogen, thus hindering tissue colonization [23]. Accompanying immune events are multiple, such as induced expression of defense response genes, callose deposition, and the oxidative burst, including hydrogen peroxide (H_2_O_2_) accumulation [24].

In the present study, we constructed a mutant of *Xoo ΔhrpE* and studied its effect on growth patterns, motility and virulence of the bacteria. Overall, in our study, we premeditated the connotation of *hrpE* in pathogen virulence and integrity as well as the functional description of *Xoo* HrpE as an elicitor of the plant immune response in rice. For this purpose, we infiltrated the plant leaves with HrpE protein produced by prokaryotic expression, and studied its roles in eliciting the HR and in inducing PTI responses. In addition, this study sheds light on the role of HrpE protein in leaf photosynthesis.

## 2. Materials and Methods

### 2.1. Bacterial Strains, Growth Conditions and Antibodies

The bacterial strains used in the present study, constructed recombinant vectors and the information about the different antibiotic resistances are given in Table S1. Different strains of *Xanthomonas oryzae* pv *oryzae* (*Xoo*) were grown on nutrient broth (NB) or nutrient agar (NA) medium at 28 °C [25]. Luria–Bertani broth (LB) or LB agar (LA) was used to culture engineered *Escherichia coli* strains on media supplemented with 100 μg/mL spectinomycin, or 50 μg/mL kanamycin or 100 μg/mL ampicillin [26].

### 2.2. Bacterial Gene Alterations

The *hrpE* gene was knocked-out from *Xoo* PXO99^A^ by adopting the marker-less deletion method [12]. Four hundred base pairs (bp) upstream and 400 bp downstream of the *hrpE* gene, with flanking partial sequence fragments, were amplified from the PXO99^A^. The amplified e*GFP* gene fragment also contained overhang sequences of up and down stream of the *hrpE* gene. These fragments were then joined to each other by overlapped fusion-PCR using fragment-specific primers listed in Table S2. Gel electrophoresis and sequencing was done to confirm each PCR product. The fragments were then cloned into the vector pK18sacB by restriction digestion using *Xba*I ((Takara Bio, Beijing, China), and *BamH*I ((Takara Bio, Beijing, China). Ligation was done by using the T4 Ligation system (Thermo Scientific, Wilmington, DE, USA) and the recombinant vector was then transformed in competent cells of DH5α ((Takara Bio, Beijing, China). Then, electroporation was done to transform the recombinant vector carrying the cloned fragment into PXO99^A^ competent cells. Single-colonies were selected from kanamycin-containing and sugar-absent NA plates. The single crossover colonies; semi-integrated into the Xoo genome were shifted to NB liquid media, grown at 28 °C for 12 h and then transferred onto NA plates containing 10% sucrose. The colonies able to grow on sucrose-supplemented media were streaked onto NA plates with and without kanamycin. This double crossover event resulted in selection of colonies showing kanamycin-negative and sucrose-positive traits, and again PCR amplification was done for the confirmation of unmarked mutants.

The protocol for construction of the cya-fused gene to analyze effector translocation was as previously described [27]. Briefly, the *hrpE* gene with the *sac*I recognition site was amplified by PCR and then inserted in the sequence of *pthXo1-cya* at the *sac*I site. The recombinant sequence of *pthXo1-cya* was inserted into *ΔhrpE* by electroporation. The complementation strain (*ΔhrpE/hrpE-pthXo1-cya*) was formed by inserting the *hrpE-pthXo1-cya* gene into the competent cell of *ΔhrpE* by electroporation. 

### 2.3. Growth Curves

A single colony of *Xoo* (PXO99^A^), three independent *ΔhrpE* mutants (*ΔhrpEI, ΔhrpEII*, and *ΔhrpE III* to achieve accuracy) and their complementation strain (*ΔhrpE/hrpE*) were cultured at 28 °C in NB medium with shaking at 200 rpm overnight. Optical density (OD_600_) of the cultures was measured, set as equal for all the strains and then transferred to new NB medium, and bacterial growth was measured at 0, 8, 16, 24, and 32 h. The colony forming units (CFUs) of the bacterial strains were analyzed by spreading the serial dilution of the bacterial cultures on NA plates. 

### 2.4. Bacterial Motility Assays

The cellular motility of PXO99^A^, *ΔhrpE* and *ΔhrpE/hrpE* was assessed by using the cultures with different concentrations of agar in NA media. OD_600_ was normalized to 1. The NA media was supplemented with different concentrations of agar (for swarming 0.7%, for twitching 1.6% and for swimming motility 0.2% agar was used) [28] and spotted with 2 µL of bacterial culture for analyzing twitching and swarming motility. A sterile tooth pick was dipped in bacterial culture and tinged gently in the center of the plate containing NA media to check the swimming. The plates were analyzed for swimming and swarming motility after 24 h of incubation at 28 ℃, whereas the twitching was observed at 48-hour post-incubation. The results were confirmed by repeating the same experiment three times with five replicates for each treatment.

### 2.5. In-Planta Growth Asssay

The germination of rice seeds was carried out in plastic trays filled with a mixture of vermiculite, sand, and peat (1:1:1 v/v/v). The germinated seedlings were transferred to 12-L pots (3 plants/pot) after 3 days. These pots were pre-filled with soil from a local rice field. The seed germination and plant growth was undertaken in temperature-controlled growth chambers at 28 °C, relative humidity of 85%, and 12-h light at 250 ± 50 μmol quanta/m^2^/sec. The tobacco plants were used after growing for 2 months in a greenhouse at 25 °C [20].

### 2.6. Bacterial Virulence Evaluation

The inoculum suspensions of PXO99^A^, *ΔhrpE* and *ΔhrpE/hrpE* were prepared with an OD_600_ = 0.5 by washing the cultures twice and re-suspending in autoclaved water, while sterilized double distilled water was used as a control in this experiment. Leaves of 2-month-old rice plants were inoculated with bacterial suspension by using the leaf-clip method [25]. The disease symptoms in all treatments were recorded by means of the leaf lesion length after 15 days post-inoculation. Hypersensitive response (HR) in tobacco leaves was observed at the inoculated sites 34-hour post-inoculation. For each treatment, the growth of bacteria was measured from infiltrated rice leaves as log cfu/leaf 15 days post leaf-center-infiltration inoculations [27].

### 2.7. Translocation Assays

Two-week-old rice seedlings inoculated with *pthXo1-Cya* transformed strains of *Xoo* were used for studying the cya reporter assay. The bacterial culture grown in NB media (OD_600_ = 0.5) was infiltrated into three sites/leaf. The leaf from the infiltrated site were cut 12 h post inoculation (hpi), ground to fine powder in liquid nitrogen by using pestle and mortar. The ground sample was suspended in 350 μL of 0.1 M HCl and centrifuged [27]. A cAMP ELISA detection kit (GenScript, Piscataway, NJ, USA) was used to analyze the obtained supernatant for determination of intracellular cAMP concentrations [26,27].

### 2.8. Expression and Purification of Recombinant Proteins

The primers phrpE-f-phrpE-r (Table S2) were used to amplify the full-length *hrpE* gene PCR from PXO99^A^ genomic DNA. Similarly, the gene encoding red fluorescent protein (RFP) was amplified by using PCR primers pRFPf and pRFPr (Table S2). The pET30a vector (Novagen®, St. Louis, USA) digested by *Bam*HI and *Hind*III restriction enzymes was used to clone the amplified products. Colony PCR and sequencing was used to confirm the transformants. The recombinant vector was then transformed into *E. coli* strain BL21(DE3) pLysS and 0.5 mM isopropyl-β-d-1-thiogalactopyranoside (IPTG) was used for 5 h at 37 °C to induce the expression of recombinant protein. The soluble portion of bacterial lysate was subjected to affinity chromatography for the purification of protein. The Ni^2+^-nitrilotriacetate (Ni-NTA) agarose column (Qiagen, Hilden, Germany) was used to purify the recombinant proteins HrpE-RFP-His (HrpE) and RFP-His (RFP) and PBS buffer was used to dialyze the proteins for 24 h. The quantification of purified proteins was done by using a BCA protein assay kit (TransGen Biotech, Beijing, China) [29].

### 2.9. Callose Staining

The callose of rice leaves was stained on the infiltration site with aniline blue and then the samples were cytologically observed by using UV fluorescence microscopy. The leaves of rice were infiltrated with 2.0 µM of HrpE, while RFP protein served as control in this study, and the callose staining for leaves of each treatment was observed by using UV fluorescence microscopy 24 hpi as reported [30]. Image J software was used to estimate the callose intensity from digital photographs by the number of blue pixels as compared to the total number of pixels covering the leaves. The presented results are relative to the callose intensity of control treatments, which was considered to be one for this study [31]. 

### 2.10. Accumulation of H_2_O_2_

The accumulation of H_2_O_2_ in rice leaves was detected by infiltrating the samples with 2.0 µM of HrpE, and RFP proteins. After 24 h post inoculation, H_2_O_2_ accumulation was visualized after staining the leaves with DAB (Sigma, St Louis, MO, USA) as reported by [32]. An optical microscope was used to observe and photograph the stained leaves. The accumulation of H_2_O_2_ was observed as the intensity of DAB calculated from the captured images by counting the brown pixels as reported [24].

### 2.11. Xoo Growth in Rice Leaves Pretreated with HrpE

The growth of *Xoo* in leaves of the rice plants infiltrated with HrpE and RFP proteins at the concentration of 2 µM and 15 mM NaCl was observed at 24 hpi after infiltrating these pre-treated leaves with 10^6^ cfu/mL *Xoo* culture by using sterile syringes. Treatment with RFP and 15 mM NaCl served as a control. The infection caused by *Xoo* was observed on the leaves at 2, 4 and 8 days post inoculation (dpi). Leaf discs of 0.5 cm diameter from each sample were ground in 1 ml of 15 mM NaCl to perform bacterial growth assays for each time interval and the suspension was plated onto NA following serial dilutions. The results are presented as log cfu/cm^2^ of leaf tissue after counting the number of colonies 48 h post-incubation at 28 °C.

### 2.12. Expression Analysis of Defense-Related Genes in Rice

Two-week-old rice plants were treated with 2.0 µM of HrpE or RFP and the samples were collected after 24 h. TRIzol^®^ reagent (Invitrogen Biotechnology Co., Carlsbad, CA, U.S.A.) was used for the extraction of total RNA from treated leaves and subjected to DNase I (Invitrogen) treatment for removing DNA as described [27]. The synthesis of first-strand cDNA was carried out by using RT enzymes (TaKaRa Bio, Beijing, China). The SYBR Premix Ex-Taq kit (TaKaRa) was used for performing quantitative PCR on the Quant studio 6.0 real-time PCR system (Applied Biosystems, CA, USA) by using specific oligonucleotide primers listed in Table S2. The genes analyzed for differential expression were OsGST (Glutathione-S-Transferase), OsSOD (Superoxide Dismutase), OsMKK4 Kinase 4 (Mitogen Activated Protein Kinase), OsPR1 and OsPR4 (Pathogenesis Related 1 and 4), OsHMGR (3-Hydroxy-Methylglutaryl CoA Reductase) and OsPAL gene encoding Phenylalanine Ammonia Lyase. The obtained values were generated as means of three biological replicates and three technical replicates for each sample as reported by [26]. The fold change or relative expression was calculated based on the comparative CT method [33].

### 2.13. Photosynthetic Activity, Transpiration Rate and Stomatal Conductance of the Rice Plant

The photosynthetic parameters were observed by treating the two-week-old rice plants via spraying with 2.0 µM of HrpE protein for 24 h followed by inoculation of the PXO99^A^ strain (HrpE P.T.). The rice plants treated with PXO99^A^ and sterilized with double distilled water were used as controls. The time frame study for consumption of CO_2_ as well as light usage depicting photosynthetic activity, transpiration rate and stomatal conductance of rice plants was performed by using the readings from seven maximum light exposed leaves from three plants of each treatment post inoculation (at an interval of 2, 4 and 6 dpi) by using an open IRGA LI-COR 6400 XT portable photosynthesis system (LI-6400, Li-Cor Inc., Lincoln, NE, USA) [34]. All parameters under study were recorded under light saturated conditions at 380 mol mol^−1^ CO_2_ concentration and a photosynthetic photon flux density of 1000 mmol photons m^−2^ s^−1^.

### 2.14. Statistical Analysis

The statistical analysis for all experiments was based on a completely randomized design. The SPSS statistical package was used for statistical analysis, Tukey’s HSD test was applied following ANOVA, and standard deviation from the means was taken at *p* ≤ 0.05.

## 3. Results

### 3.1. HrpE Gene Deletion Affects the Growth and Colony Forming Units (CFUs)

**Figure 1:**
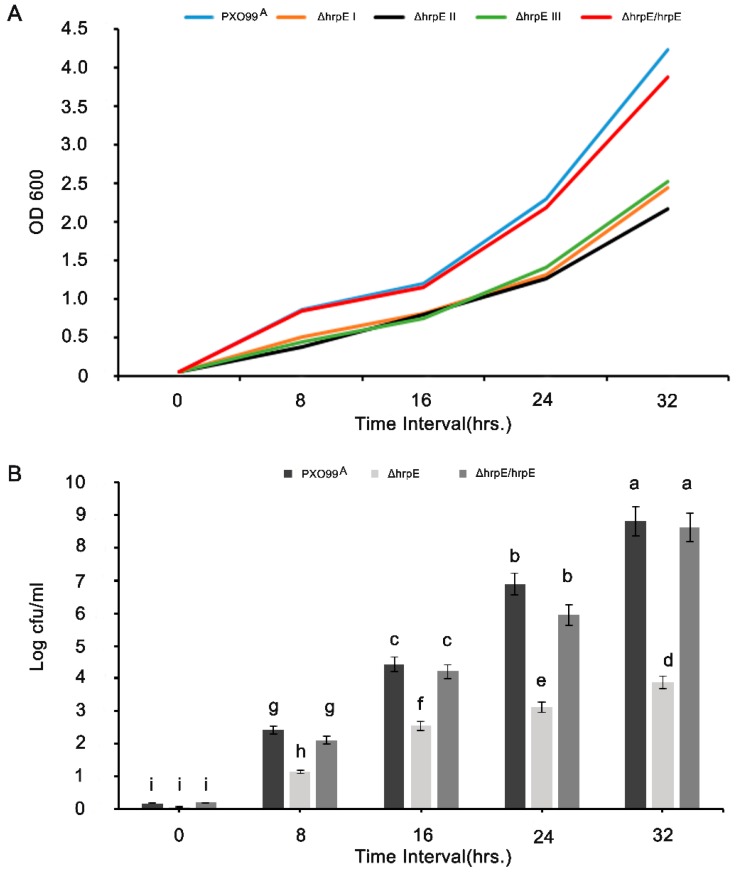
Growth curves of three independent *Xoo ΔhrpE* colonies in comparison with WT *Xoo* (PXO99^A^) at different time intervals; the complementation strain *Xoo* (*ΔhrpE/hrpE*) served as a control (**A**). Quantification of the log colony forming unit (log cfu/mL) of different bacterial strains on NA media (**B**). The error bars on the graph indicate standard deviations. Different alphabets on the bars describe significant differences among the treatments at *p* < 0.05. The experiment was conducted three times with similar results.

To analyze the function of HrpE, the PXO99^A^ mutant *ΔhrpE* was used. The mutations were confirmed by PCR (Figure S1). The *ΔhrpE* mutants grew slower than PXO99^A^ and the complementation strain (Figure 1A). The delayed growth of the *ΔhrpE* mutant was recovered by the introduction of *hrpE* back into the mutant, demonstrating that *hrpE* deletion affects bacterial growth. The time-interval study for evaluating the colony forming units (CFU) suggested that the *hrpE* deletion mutant had the lowest CFU as compared to the other strains (Figure 1B). The growth patterns and CFU of all strains indicated that *hrpE* is involved in regulation of bacterial growth.

### 3.2. Deletion of hrpE Affects the Bacterial Motility

**Figure 2:**
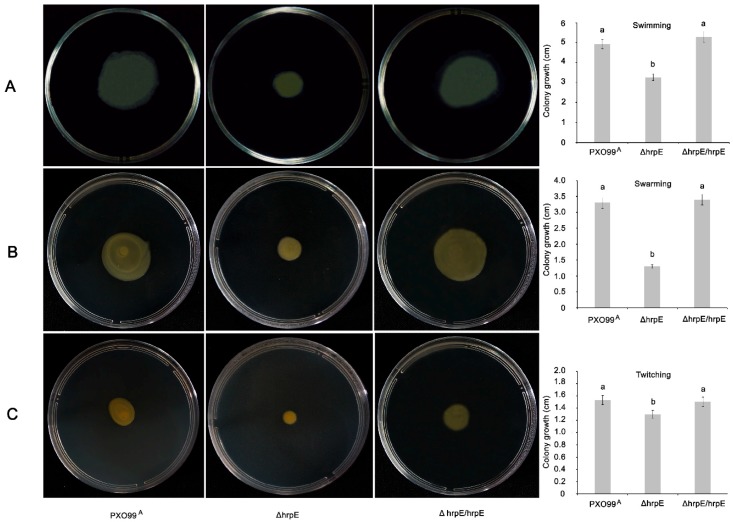
Effect of *hrpE* deletion on swimming, swarming and twitching motility. Swimming motility of different bacterial strains recorded 24 h after inoculation (**A**), swarming motility of different bacterial strains recorded 24 h after inoculation (**B**) and twitching motility of bacterial strains recorded 48 h after inoculation (**C**). Error bars in graph indicate standard deviations of five replicates. Different letters on the bars describe significant differences at *p* < 0.05. All experiments were repeated thrice with similar results each time.

The *ΔhrpE* mutants showed significantly less growth in NA media. All three types of motility were affected in the *ΔhrpE* mutant compared to the wild-type strain PXO99^A^ (Figure 2). Swimming motility was constrained in *ΔhrpE* compared to the PXO99^A^, and a significantly smaller diameter was recorded in *ΔhrpE* than that in WT (wild type) PXO99^A^ and *ΔhrpE/hrpE* complementation strains (Figure 2A). Swarming motility was also reduced to ½-fold of PXO99^A^ and *ΔhrpE/hrpE* strains (Figure 2B). Moreover, twitching motility was most affected in the *ΔhrpE* mutant (Figure 2C).

### 3.3. Deletion of hrpE Affects the Pathogen Virulence in a Host and the Hypersensitive Response in a Non-Host

**Figure 3:**
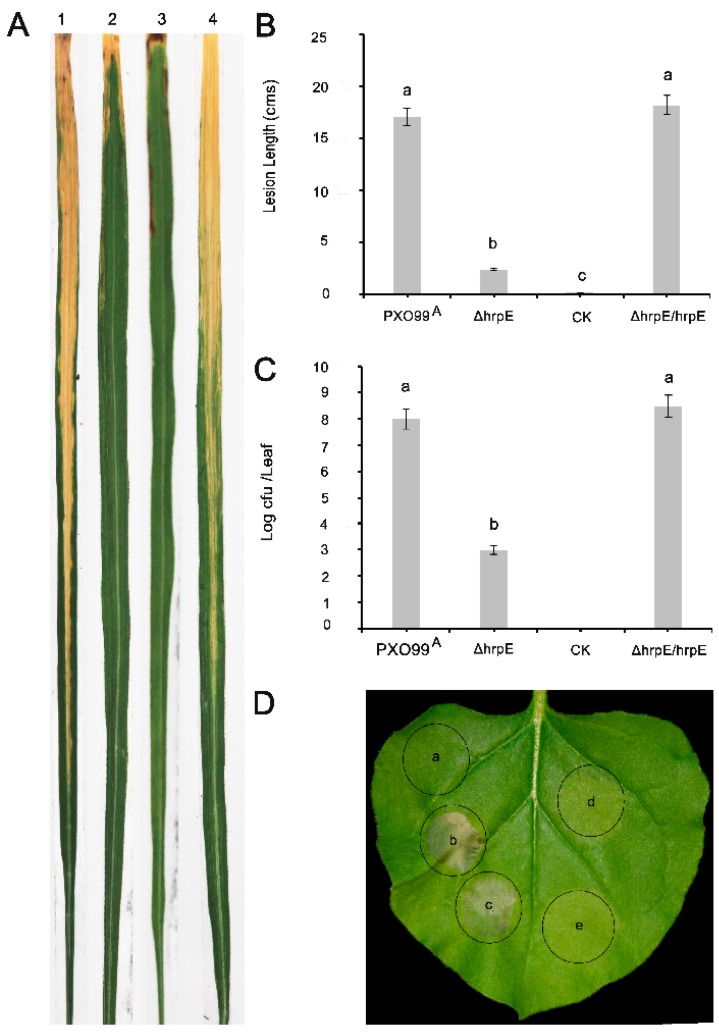
Deletion of the *hrpE* gene reduces the virulence of *Xoo* strain PXO99^A^ in Nipponbare. Symptoms of the bacterial blight on rice leaves after 15 days of leaf-top-clipping on Nipponbare rice; 1. PXO99^A^ 2. *ΔhrpE* 3. CK (water) 4. *ΔhrpE/hrpE* (**A**). Lesion length of bacterial blight symptoms of the leaves from A (**B**). Bacterial population count presented as CFU (colony formation unit) of bacterial cultures from rice leaves after 15 days of leaf-center inoculations (**C**). Hypersensitive response in tobacco leaves after 34 h of inoculation against *Xoo* strains; (a) Ck (Sterilized double distilled Water), (b) PXO99^A^, (c) *ΔhrpE/hrpE*, (d) *ΔhrpE*, (e)15 mM NaCl (**D**). Error bars on graph shows standard deviations. Values are presented as the mean of three replicates. Different letters on the bars in the graph states significant differences at *p* < 0.05. The experiments were repeated thrice with similar results each time.

The leaves inoculated with *ΔhrpE* showed significantly less symptoms compared to those inoculated with PXO99^A^ (Figure 3A). The lesion length was 2.1 cm in *ΔhrpE*-inoculated leaves, whereas it was 16.9 cm in the wild type strain (Figure 3B). The *ΔhrpE/hrpE* produced symptoms on rice similar to PXO99^A^, suggesting that *hrpE* deletion affects PXO99^A^ virulence. To further confirm this result, bacterial populations were measured. The *ΔhrpE* mutant multiplied slower in rice leaves compared to PXO99^A^ and *ΔhrpE/hrpE* (Figure 3C). These results demonstrated that *hrpE* is involved in PXO99^A^ virulence.

To evaluate whether HrpE is able to elicit HR, *Nicotiana benthamiana* leaves were infiltrated with *ΔhrpE*, PXO99^A^, and *ΔhrpE/hrpE.* Tobacco leaves infiltrated with PXO99^A^ and *ΔhrpE/hrpE* showed HR, while *ΔhrpE* failed to elicit HR (Figure 3D), indicating that HrpE induces HR in *N. benthamiana*.

### 3.4. HrpE Pilus Serves as Conduit for Effector Translocation

**Figure 4:**
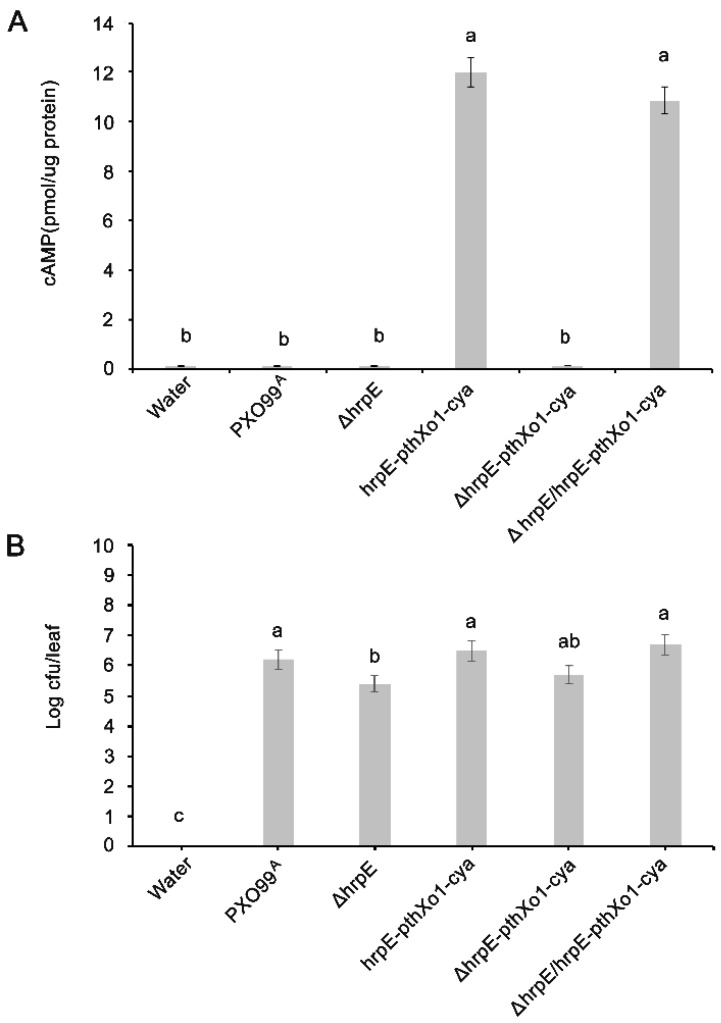
Concentrations of cAMP in leaves of Nipponbare rice plants 12 hpi with different strains of *Xoo* (**A**). The CFU of inoculated bacteria in rice leaves 12 hpi with different *Xoo* strains (**B**). Error bars on the graph represents standard deviations of three replicates. Different letters refer to significant differences at *p* < 0.05. The experiments were repeated three times with similar results.

Leaves were collected 12 h after infiltration, and translocation of the effectors was measured by quantifying the concentration of cAMP in the leaf cells. Higher levels of pthXo1 translocation was detected from *hrpE-pthXo1-cya* bacteria at 12 hpi, whereas cAMP concentration was significantly decreased (*p* < 0.05) in *ΔhrpE-pthXo1-cya* in which *hrpE* is deleted (Figure 4A). However, cya activity was increased when the leaves were infiltrated with *ΔhrpE/hrpE-pthXo1-cya* bacteria showing the higher concentrations of cAMP indicating that complementing the mutant strain recovered its ability to translocate the effector pthXo1. Bacterial strains without *pthXo1*-*cya* served as controls. There was no significant difference in the CFU of all bacterial treatments (Figure 4B), this indicates the reduced cya activity in *ΔhrpE-pthXo1-cya* was mainly caused by efficiency of effector translocation and not by growth reduction. These results demonstrate that HrpE is involved in effector translocation.

### 3.5. Defense Responses Triggered by HrpE Protein

**Figure 5:**
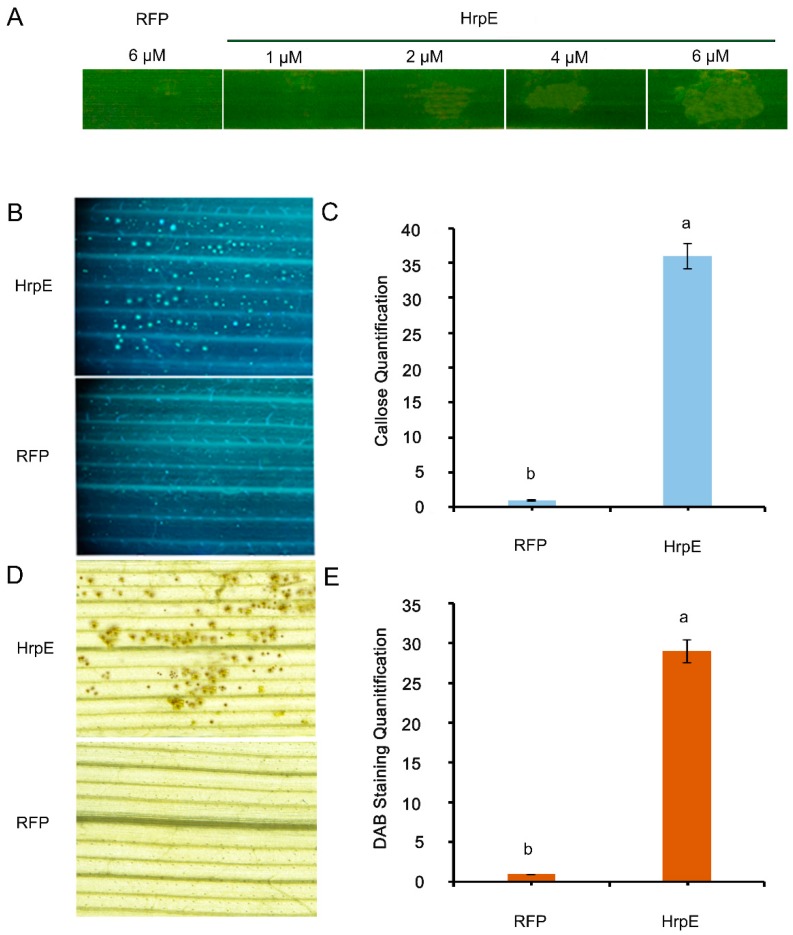
Analysis of rice leaf response to *Xoo* HrpE. Leaf response to infiltration of purified HrpE at different concentrations and 6 µM RFP as a control 24 hpi (**A**). Photographs representing the deposition of callose in rice leaves center-infiltrated with HrpE, with RFP as a control (**B**). Quantification of callose deposition intensity in rice leaves (**C**). Representative photographs for H_2_O_2_ accumulation in leaves of rice infiltrated with HrpE, with RFP as a control (**D**). Quantification of H_2_O_2_ accumulation in rice leaves by DAB staining in rice leaves (**E**). For callose quantification and DAB staining quantification, values are the mean calculated from 10 different photographs. Each experiment was repeated three times. Error bars represent standard deviations. Different letters on the graph bars represent significant differences among the treatments at *p* < 0.05.

To evaluate if *Xoo* HrpE could elicit defense responses in rice, recombinant HrpE-RFP-His (HrpE) protein was purified from *E. coli*. Western blot analysis validated the degree of purity and reliability of the recombinant proteins (Figure S2), and RFP was used as a negative control. Rice leaves were infiltrated with different concentrations of the recombinant proteins. The infiltrated leaves showed chlorotic lesions 24 h after infiltration when HrpE protein concentration was increased to 2 µM. The intensity of chlorotic lesions on the leaves were directly proportional to the dose of the HrpE protein, whereas the leaves infiltrated with the RFP showed no obvious lesions (Figure 5A). At 24 hpi, rice leaves showed significant deposition of callose, while control leaves showed less callose deposition (Figure 5B,C). Similarly, H_2_O_2_ accumulation was significantly higher in the leaves infiltrated with HrpE than in the control leaves treated with RFP (Figure 5D,E).

### 3.6. Pre-Treatment of Rice Leaves with HrpE Supresses Xanthomonas oryzae pv oryzae (Xoo) Growth

**Figure 6:**
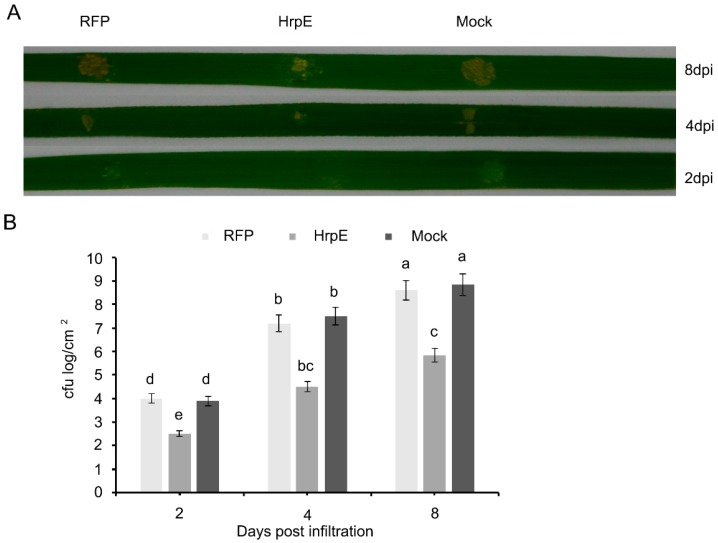
*Xoo* infections in rice leaves pre-infiltrated with HrpE, at 2, 4 and 8 days post infiltration (dpi) where RFP and Mock (15 mM NaCl) served as a control (**A**). Calculation of log bacterial population in the leaves of rice plants at 2,4 and 8 dpi (**B**). Error bars show the standard deviations of the means calculated from three different replicates. Lower case letters in the graph bars represent a significant difference at *p* < 0.05. Experiments were conducted thrice with similar results each time.

HrpE pre-treatment promotes plant resistance against *Xoo*. The leaves were infiltrated with 2 µM of HrpE or RFP recombinant protein. Later, the infiltrated leaves were inoculated with PXO99^A^ 24 h after treatment of the recombinant protein. The disease symptoms in HrpE-pretreated leaves were significantly reduced compared to controls (Figure 6A). The bacterial population measured as cfu log was significantly less in the leaves pre-treated with HrpE compared to RFP and mock controls (Figure 6B).

### 3.7. Expression Profiling of the Plant Defense Related Genes under the Influence of HrpE

**Figure 7:**
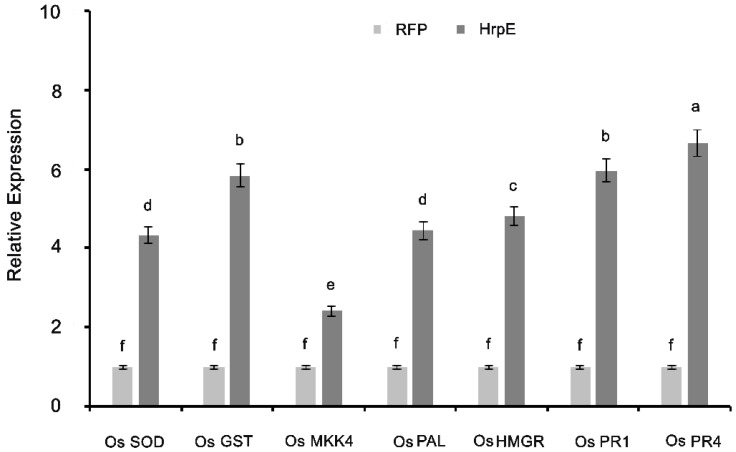
Expression profiling of the rice defense-related genes in the leaves infiltrated with HrpE by q RT-PCR. Values represent the average of three biological replicates with three technical replicates. Error bars signify standard deviations of the means. Different letters above the columns in the graph show a significant difference among the treatments at *p* < 0.05. The experiment was repeated three times.

The genes analyzed for differential expression were *OsGST* (Glutathione-S-Transferase), *OsSOD* (Superoxide Dismutase), *OsMKK4* Kinase 4 (Mitogen Activated Protein Kinase), *OsPR1* and *OsPR4* (Pathogenesis Related 1 and 4), *OsHMGR* (3-Hydroxy-Methylglutaryl CoA Reductase) and *OsPAL* gene encoding Phenylalanine Ammonia Lyase. In HrpE infiltrated rice plants, there was a significant increase in the expression levels of all the genes under study as compared to the rice plants treated with the RFP protein as a control (*p* < 0.05) (Figure 7).

### 3.8. Pre-Treatment with HrpE Influences the Photosynthetic Activity, Transpiration Rate and Stomatal Conductance of the Rice Plant

**Figure 8:**
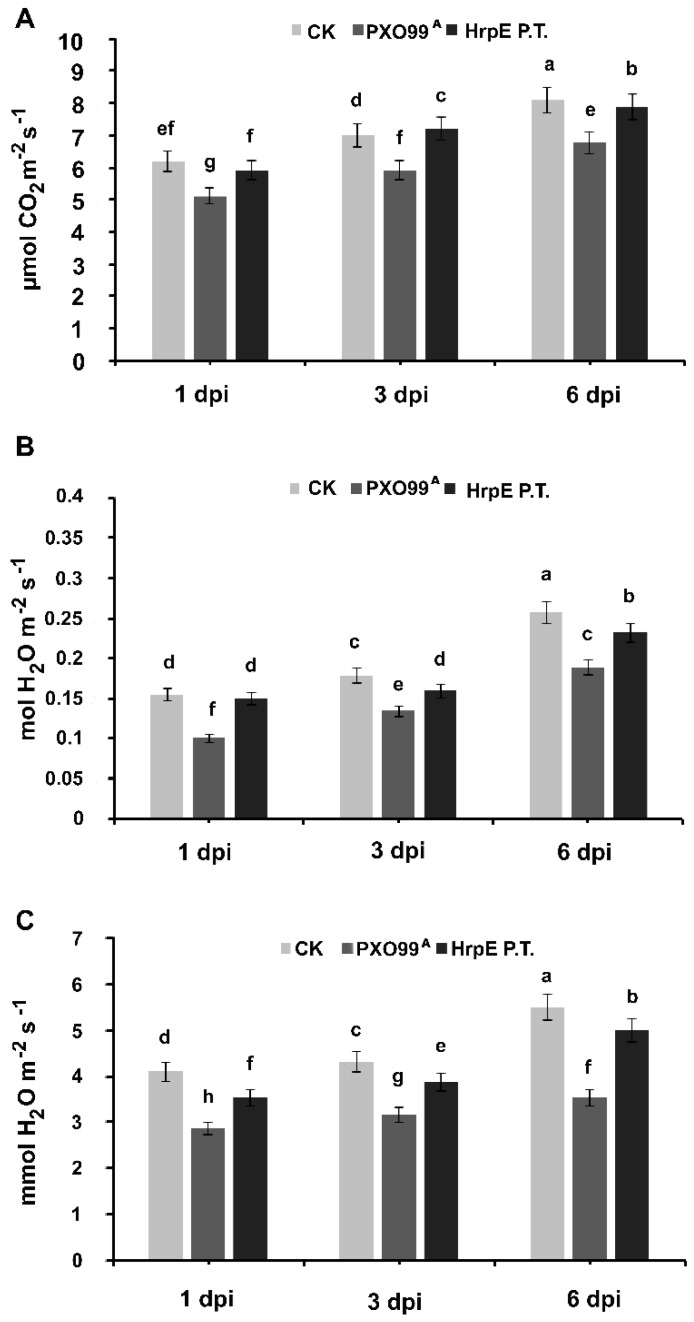
Photosynthesis rate of rice leaves pre-treated with HrpE at different time intervals (**A**); stomatal conductance (**B**); and transpiration rate (**C**). dpi = days post inoculation. Error bars show standard deviations of the means. Lower case letters above the columns indicate a significant difference at *p* < 0.05. All the experiments were conducted in triplicate with similar results each time.

Rice leaves were pre-treated with HrpE or RFP proteins, and then inoculated with PXO99^A^, and leaves pre-treated with water alone were used as a control. Photosynthetic efficiency of the plants was determined at different time intervals. Significant increases in the stomatal conductance (Figure 8B), photosynthetic activity (Figure 8A) and transpiration rate (Figure 8C) were observed in PXO99^A^ infected leaves pre-treated with the HrpE compared with those inoculated with PXO99^A^ alone, which is comparable to those treated with water alone.

## 4. Discussion 

In *Xanthomonas*, the HrpE protein has been identified as a major structural component required for pilus formation [35]. The protein sequence analysis showed that the *Xoo* HrpE protein is differentiated from other *Xanthomonas* species based on sequence dissimilarity (Figure S3). Bacterial effectors are translocated into plant cytosol by the T3 secretory pilus, causing disease in host plants [27]. Based on the already established facts about the critical role of HrpE in plant disease onset, in the present study we constructed a *hrpE* gene deletion mutant of *Xoo* (PXO99^A^) and analyzed the effect of this deletion on the physiological parameters related to the growth and motility of the bacteria and its virulence. 

In order to develop plant–pathogen interactions, bacteria use their pili or fimbriae to communicate and interact with each other, as well as with plants [9]. In this study, the deletion of the *hrpE* gene resulted in a significantly lower growth rate of *Xoo* ΔhrpE as compared to PXO99^A^ and the complementation strain. To the best of our knowledge, there are no studies reporting the effect of the hrp gene deletion on bacterial growth and the underlying mechanism is still unknown. However, there are certain reports that demonstrate that deletion of the AS87-03730 gene associated with virulence may negatively affect bacterial growth in liquid media [11]. The gram-negative bacterium, *Xoo*, is capable of three different types of motility: swimming, swarming and twitching [5]. The results from our study demonstrated that bacterial motility was significantly reduced by *hrpE* deletion, which is in accordance with a previous study stating that the deletion of the pilin-like gene *pilA* negatively affected the motility of *Xanthomonas citri* [36]. Overall, our results showed that deletion of *hrpE* reduced the growth of *Xoo* in liquid media, as well as its motility.

Apart from affecting the growth and motility, *hrpE* deletion resulted in disruption in the translocation of effectors, which are extremely essential for disease onset in the plant. Several *hrp* genes that are responsible for translocating effectors, can also induce HR in non-host plants [37]. The present study indicated that the *hrpE* mutant was deficient in both HR elicitations in a non-host plant (*Nicotiana benthamiana*) and pathogenicity in a host plant (Nipponbare rice). ΔhrpE failed to translocate the pathogenesis-linked effectors and other toxic proteins into host cells and resulted in a decrease, or almost failure, of disease. This demonstrates that in addition to having an essential role in effector translocation, HrpE also serves as one of the key virulence factors. A complete loss of pathogenicity occurred when any of the hrp genes were mutated [38]. The current study highlights that the translocation of the TAL effector pthXol was blocked in *Xoo* ΔhrpE based on the Cya reporter assay. Therefore, we can conclude that HrpE functions as a type III translocator for effector translocation. Our findings are supported by a previous study stating that the harpin protein Hpa1 functions in mediating effector translocation from bacterial cells to the cytosol of the rice plant [27].

Additionally, harpins have been considered as activators of the plant immune response and confer plant disease resistance through induced systemic resistance pathways [18]. Hpa1 protein from *Xoo* plays an important role in induction of defense response in rice plants and it has been illustrated to affect disease resistance in *Arabidopsis* [21]. In our study, we used purified HrpE protein to elicit a defense response in rice plants. Rice leaves infiltrated with the HrpE protein resulted in activation of various defense responses such as developing visible leaf lesions, enhancing callose deposition and promoting H_2_O_2_ accumulation in rice leaf tissues. Pathogen challenged rice leaves pre-infiltrated with HrpE protein resulted in significantly reduced bacterial growth compared to the mock and RFP treated control, further confirming that HrpE functions as an elicitor of the defense response. HrpE from *Xcc* has been reported to initiate the expression of defense-related genes [31]. Similarly, infiltration of *Xoo* HrpE induced systemic resistance in rice by modulating the expression of various defense-related genes. 

Plants are highly sensitive to stress and respond either by inducing systemic resistance or by affecting plant growth through altering metabolic activities [39,40]. Pre-infiltration of the rice plants with HrpE protein activated their defense response. When pre-infiltrated rice plants were challenged with *Xoo*, fluctuation occurred in various physiological activities of the plants. The photosynthetic parameters of the plants were reduced in the infected control leaves, while the plants pre-infiltrated with the HrpE protein were already primed to tackle the pathogen; hence, the photosynthetic efficiency was also enhanced in pathogen challenged plants. These results indicate that HrpE treatment significantly improves the plant photosynthetic parameters. Hpa1 has been reported to promote the vegetative growth of the plants and increase their photosynthetic efficiency [41], which is in concordance with our findings.

The significance of this study is highlighted by the fact that *hrpE* gene deletion affected the bacterial growth patterns and motility. The gene deletion impaired the secretion of T3 effectors, and thus affected pathogen virulence. Our study also demonstrated that plants have developed a capacity to identify the HrpE protein as an immune signal, enabling them to protect themselves against pathogens and aiding in enhanced photosynthetic efficiency (Figure S4). Based on the potential of this protein to elicit the defense response, it is highly desirable to investigate the transgenic expression of this protein in plants, aiding the development of resistant rice cultivars.

## Supplementary Materials

The following are available online at https://www.mdpi.com/2076-2607/7/11/572/s1.
