# Supplementary material for: The Type III Accessory Protein HrpE of Xanthomonas oryzae pv. oryzae Surpasses the Secretion Role, and Enhances Plant Resistance and Photosynthesis
Source: Microorganisms. 2019 Nov 18;7(11):572. doi: 10.3390/microorganisms7110572 (PMC6921029; doi:10.3390/microorganisms7110572)
Supplement: Supplementary file 1 [file microorganisms-07-00572-s001.pdf]

Supplementary data

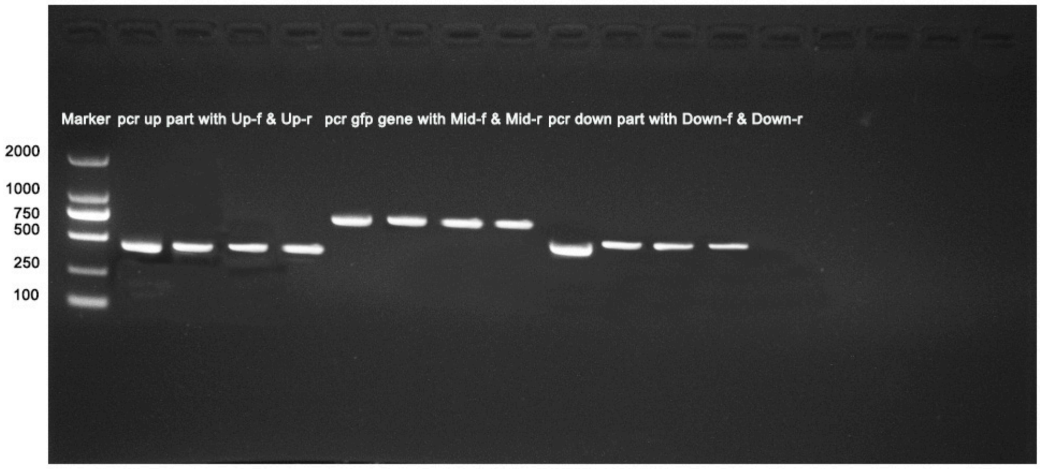

Figure S1. PCR of bacterial colonies with primers of up mid and down gene confirms the mutant formation.

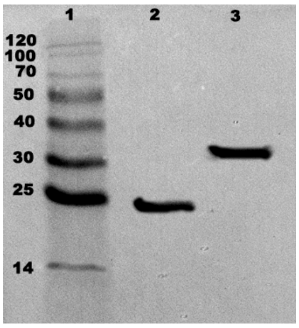

Figure S2. Western blot Analysis of RFP-His (RFP) and HrpE-RFP-His (HrpE) purified protein.

Lane 1 represents the ladder of 120 kDa protein marker.

Lane 2 represents the RFP protein

Lane 3 represents the HrpE protein

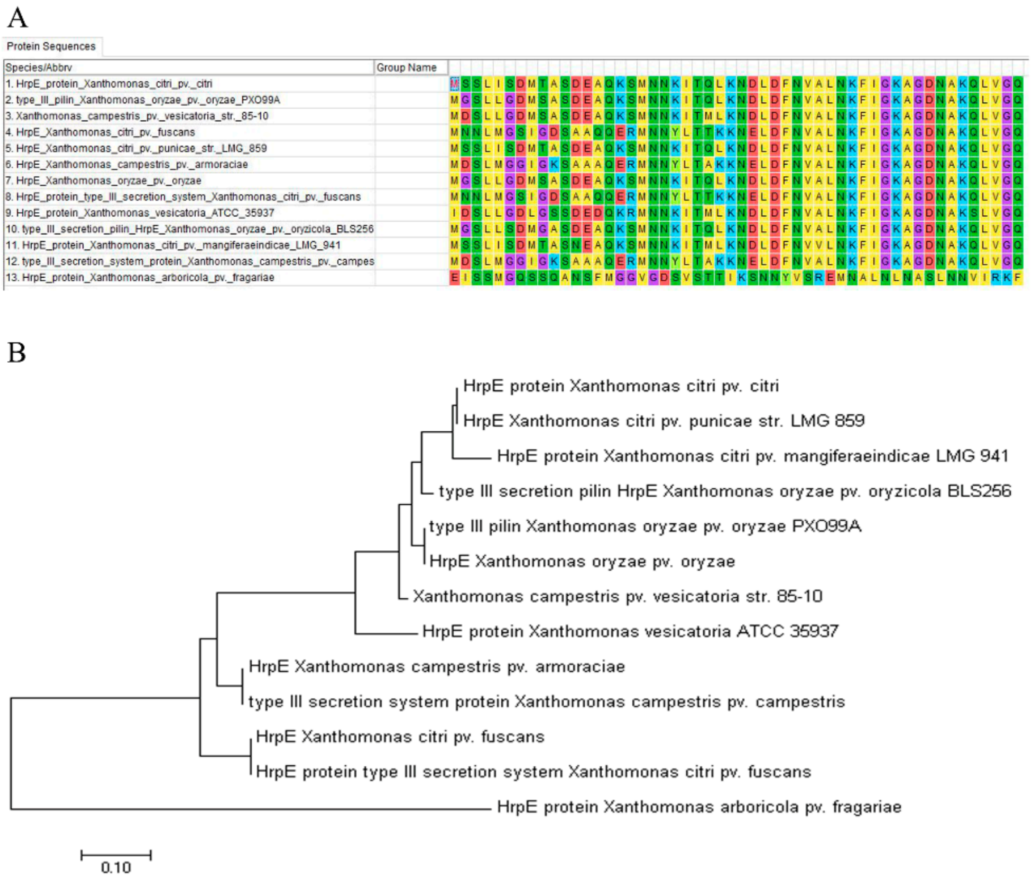

Figure S3. Phylogenetic relationship in different species of *Xanthomonas*. The sequence of the *HrpE* protein in different species of *Xanthomonas* was aligned using ClustalW program (A). The phylogenetic relationships of 13 different species and strains of *Xanthomonas* genus obtained using MEGA 7.0 with a maximum likelihood approach (B).

Table S1

Strains and Plasmids	Relevant characteristics	Source/Reference
Escherichia coli DH5α	F^- 80dlacZ M15(lacZYA-argF) U169 endA1 deoR recA1 hsdR17(r_K^- m_K^+) phoA supE44 λ^- thi-l gyrA96 relA1	This lab
BL21(DE3)	F^- *ompT hsdSB* (r_B^- m_B^-) *gal dcm* (DE3)	Novagen
Xanthomonas oryzae pv. oryzae PXO99^A	Philippine race 6; azacytidine resistant clone of PXO99^A, virulent to rice cultivars Nipponbare and IRBB10	This lab
ΔhrpE	PXO99^A *HrpE* deletion mutant	This study

ΔhrpE/HrpE	PXO99[A] *HrpE* mutant complemented with pHM*HrpE*	This study
HrpE-pthXo1-cya	PXO99[A] *HrpE* transformed with pHMpthXo1-cya	This study
ΔhrpE-pthXo1-cya	PXO99[A] *HrpE* mutant transformed with pHMpthXo1-cya	This study
ΔhrpE/HrpE/pthXo1-cya	PXO99[A] *HrpE* mutant complemented with pHMHrpE-pthXo1-cya	This study
pMD-19	Vector	Takara
pMD-18	Vector	Takara
pK18*sacB*	Suicide vector derivative from pK18mobGII, *sacB*$^{+}$, Km^{R}	This lab
pHM1	Broad-host range vector with pUC19 polylinker, Sp^{R}	This lab
pZW*pthXo1*	PthXo1 fused to *lacZ* promoter of pBluescript II KS(+)	Yang et al., 2006
pZW*pthXo1-cya*	Cya tag inserted in the *Sac* I site of pZW*pthXo1*	This lab
pMD18-T simple	pUC *ori*, cloning vector, Amp^{R}	Takara
pET30a (+)	pBR322 origin, *T7* promoter His-tag, Km^{R}	Novogen

Table S 2

**Oligonucleotides**	**Sequence**	**Use**
HrpE-f	CCCAAGCTTATGGAAATACTTCCGCAAA	Full *HrpE* cloning from genomic DNA
HrpE-r	CCGGAATTCGTGATGGTGATGGTGATGGCTGGCCAACGAGCT	Full *HrpE* cloning from genomic DNA
Up-f	CGCGGATCCTGCACTATGTCGAAACGCCG	Cloning of upstream part for mutant
Up-r	TCCTCGCCCTTGCTCACCATAACAAGACTCCTACGTAGTG	Cloning of upstream part for mutant

Mid-f	CACTACGTAGGAGTCTTGTTATGGTGAGCAAGGGCGAGGA	Cloning of mid part for mutant
Mid-r	CCTGCGCATGTCTAAGCCCATTACTTGTACAGCTCGTCCA	Cloning of mid part for mutant
Down-f	TGGACGAGCTGTACAAGTAATGGGCTTAGACATGCGCAGG	Cloning of downstream part for mutant
Down-r	TGCTCTAGAACGCGCCATCCACGTCGTCG	Cloning of downstream part for mutant
egfp-f	ATGGTGAGCAAGGGCGAGGAGC	Cloning of *egfp* fragment
egfp-r	CTTGTACAGCTCGTCCATGCCGAG	Cloning of e*gfp* fragment
pHrpE-f	CGCGGATCCATGGAAATACTTCCGCAAAT	Full *HrpE* cloning in PET30
pHrpE-r	CCCAAGCTTTCACTGGCCAACGAGCT	Full *HrpE* cloning in PET30
OsSOD-f	GCTTCCACATCCACTCCTTTG	RT-qPCR
OsSOD-r	CCCCATCAATTTCACAACCTAAG	RT-qPCR
OsMKK4-f	TCCAAAAGCGATGAGACCG	RT-qPCR
OsMKK4-r	AAGTAGGTGGGAGAGGAAGAG	RT-qPCR
OsPAL-f	AACTAAGCCAAGATCCCATCG	RT-qPCR
OsPAL-r	TGTCTGAAGATACACGAAGCG	RT-qPCR
OsGST-f	ATGTAATGGTGTAGCCTCACG	RT-qPCR
OsGST-r	CCTCGACTCTGCTCATTAACTC	RT-qPCR
OsHMGR-f	ACACCCAAGAGCTTCACATG	RT-qPCR
OsHMGR-r	CCTCCGCCTGGTAAATATGG	RT-qPCR
OsPR4-f	GGTGTCCGAGAAGCAGTAC	RT-qPCR
OsPR4-r	CACCTGCGTGTAGTGGC	RT-qPCR
OsPR1-f	GTGCAATGGAGTTTGTGGTC	RT-qPCR
OsPR1-r	GCTTCTCGTTCACATAATTCCC	RT-qPCR
